# Persistent Lactic Acidosis: Thinking Outside the Box

**DOI:** 10.7759/cureus.2561

**Published:** 2018-05-01

**Authors:** Hamza Arif, Sohaib Zahid, Amit Kaura

**Affiliations:** 1 Internal Medicine, Allegheny Health Network, Pittsburgh, USA; 2 Critical Care Medicine, Allegheny Health Network, Pittsburgh, USA

**Keywords:** lactic acidosis, type b lactic acidosis, diffuse large b-cell lymphoma (dlbcl)

## Abstract

A 54-year-old male presented with possible sepsis and elevated serum lactic acid (LA) of 18.7 mmol/L. Despite the sepsis treatment protocol and the management of other causes of type A lactic acidosis, his LA remained elevated. Herein, we present a case of type B lactic acidosis in the setting of a diffuse large B cell lymphoma. The proposed mechanisms of persistent lactic acidosis in malignancy are highlighted in this case report.

## Introduction

Lactic acid (LA) is an important intermediate in the metabolism of carbohydrates and non-essential amino acids [1]. When LA production exceeds its clearance, either due to marked tissue hypo-perfusion (type A) or causes other than tissue hypoxia (type B), this leads to its accumulation causing lactic acidosis [2-3]. Here, we present a case of type B lactic acidosis from a previously undiagnosed, underlying hematologic malignancy in an intensive care unit (ICU) setting.

## Case presentation

A 54-year-old male with a history of non-ischemic cardiomyopathy (left ventricular ejection fraction of 20%), atrial fibrillation, hypertension, and insulin-dependent diabetes mellitus presented with lethargy and confusion. His home medications included aspirin, glimepiride, duloxetine, gabapentin, insulin glargine, prasugrel, furosemide, sotalol, rivaroxaban, and spironolactone. He also did not have a history of tobacco, alcohol, or illicit drug use.

He had a prior hospitalization for orthostatic hypotension and was eventually discharged to a skilled nursing facility but returned to the hospital three days later with an acute change in his mental status. On presentation, he was noted to have a respiratory rate of 26 breaths per minute, a heart rate of 62 beats per minute, blood pressure of 121/73 mmHg, a temperature of 37°C, and oxygen saturation of 97% on room air. The laboratory investigation revealed thrombocytopenia of 35 k/mcL as compared to 137 k/mcL three days earlier. Further workup was significant for an anion gap of 40 and LA of 18.7 mmol/L (reference range (ref) 0.5 - 2.0 mmol/L). He had also developed acute renal injury with creatinine of 1.9 mg/dL (ref 0.70 - 1.50 mg/dL). Liver enzymes were also elevated with aspartate aminotransferase (AST) of 254 U/L (ref 17 - 59 U/L), alanine aminotransferase (ALT) of 180 U/L (ref 21 - 72 U/L), and total bilirubin of 1.9 mg/dL (ref 0.2 - 1.2 mg/dL). Sepsis bundle was initiated with intravenous (IV) fluids and broad-spectrum antibiotics as well as microbial cultures, including the collection of two sets of blood cultures.

Additional investigation showed that lactate dehydrogenase was 1472 U/L (ref 110 - 216 U/L) but haptoglobin was normal: 85.9 mg/dL (ref 16 - 200 mg/dL). His hemoglobin was 6.4 g/dL (ref 14 - 17.4 g/dL), requiring blood transfusions while he also developed leukopenia; absolute neutrophil count (ANC) was 670. Due to his change in mental status associated with thrombocytopenia, anemia, and renal injury, there was a concern for thrombotic thrombocytopenic purpura (TTP). The peripheral blood smear demonstrated decreased platelets and an absence of schistocytes or blasts, but it did show numerous smudge cells and a few nucleated blood cells. ADAMTS-13 was 98 (ref >66 %) ruling out TTP. A computed tomography (CT) scan of the chest, abdomen, and pelvis only revealed an enlarged liver and moderate splenomegaly but did not show any lymphadenopathy.

**Figure 1:**
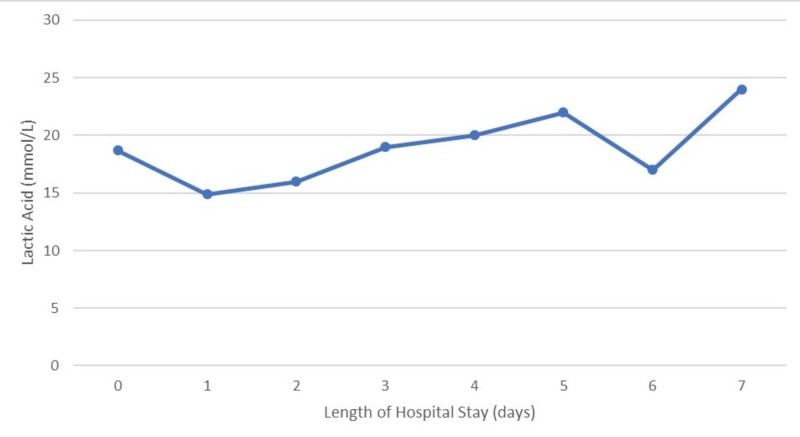
Trend of lactic acid levels during the patient's hospital stay

Although his presentation was considered likely from sepsis, as his condition continued to deteriorate, hemophagocytic lymphohistiocytosis (HLH) was also considered. He met four out of five criteria for HLH with a ferritin of 9374 ng/mL (ref 20 - 300 ng/mL) cytopenia, fever, and splenomegaly. However, his serum triglyceride was 244 mg/dL, which is lower than 265 mg/dL, the criteria needed for HLH. As the patient continued to deteriorate with worsening lactic acidosis (Figure 1) despite broad-spectrum antibiotics, thiamine administration, and continuous renal replacement therapy (CRRT), a bone marrow biopsy was performed to evaluate for marrow erythrocytic phagocytosis. He was also started on the HLH treatment protocol, receiving dexamethasone. Cyclosporine was initiated the next day, followed by etoposide. He developed multiorgan failure with persistent encephalopathy, despite discontinuing all sedation, coagulopathy, worsening liver enzymes, and cytopenia. Serum LA remained elevated with a minimum value of 14.9 mmol/L during his seven-day hospital course (Figure 1) and arterial blood pH varied from 7.15 to 7.25.

A bone marrow biopsy showed that hemophagocytosis was not identified. The bone marrow contained a monomorphic cellular infiltrate with areas of necrosis and cellular degeneration. Less than 10% of the marrow appeared fully viable and contained a monomorphous infiltrate of cells with irregular nuclear contours and pale cytoplasm. The infiltrate was positive for CD45, CD20, and CD79a, and negative for CD34, with patchy non-specific staining for TdT. The Ki67 (MIB-1) proliferation rate was up to 60%. The absence of CD34 and a convincing TdT expression favored large B-cell lymphoma. The disease was classified as Ann Arbor stage IV due to its presence in the extra-lymphatic organs (liver and bone marrow). The age-adjusted international prognostic index (IPI) score was calculated to be 3, conferring high risk.

Chemotherapy regimen “R-CHOP” for this diagnosis of diffuse large B-cell lymphoma (DLBCL) was planned with a few variations. As rituximab is dialyzed, it was not administered because he required CRRT and vincristine was held due to its hepatotoxicity on already deranged liver enzymes. Steroids were changed to IV methylprednisolone. Doxorubicin was also initiated followed by cyclophosphamide and etoposide while cyclosporine was discontinued. Unfortunately, his condition continued to deteriorate, and he became bradycardic and hypotensive, eventually requiring multiple vasopressors. After discussions with the family, comfort measures were pursued.

## Discussion

Types A and B lactic acidosis are commonly described states, with type A being the most prevalent [4]. While type A lactic acidosis is usually associated with tissue hypo-perfusion, such as mesenteric ischemia, sepsis, or shock, type B lactic acidosis can occur even when tissues are well perfused [2]. Multiple etiologies of type B lactic acidosis have been proposed (Table 1), including malignancies, particularly lymphomas, which appear to be a marker of poor prognosis [2,5].

**Table 1:** Common causes of lactic acidosis

Type A	Type B
Shock (sepsis, hypovolemic, cardiogenic)	Drugs, e.g. metformin (with high doses or renal failure), beta-agonists, nucleoside reverse transcriptase inhibitors
Cyanide toxicity, carbon monoxide poisoning	Malignancy
Severe anemia	Thiamine deficiency
	Alcoholism, liver failure
	Seizures

The association of malignancy with type B lactic acidosis has been largely limited to case reports. Some of the described malignancies include mantle cell lymphoma [6], Burkitt lymphoma [7], small cell carcinoma of the lung [8], chronic lymphocytic leukemia [9], chronic myelomonocytic leukemia [10], and multiple myeloma [11]. A case in Taiwan also reported diffuse large B-cell lymphoma (DLBCL) presenting with lactic acidosis, similar to our patient, but that patient did have bone marrow findings of HLH [12].

D-lactic acidosis has also been described, which may be observed in patients with short bowel syndrome or the ingestion of propylene glycol. However, the usual laboratory analysis does not detect D-lactate. This form of lactic acidosis should be considered in patients with a typical history and the presence of metabolic acidosis on further workup.

Multiple mechanisms have been proposed for elevated LA in malignancy, including decreased hepatic clearance of LA, thiamine deficiency, and increased production of LA by the tumor cells themselves by utilizing anaerobic glycolysis, a phenomenon referred to as the “Warburg” effect [5,13-16].

Although smudge cells are characteristically associated with chronic lymphocytic leukemia, their presence is non-specific, as they may also be seen in infections, cardiac arrest, solid malignancies, and other hematological malignancies [17].

In our patient, the causes associated with type A lactic acidosis were ruled out. He was not on any home medications associated with lactic acidosis and had no history of alcohol abuse. He did not require vasopressors during his initial presentation and his clinical condition did not improve despite multiple broad-spectrum antibiotics, ruling out both cardiogenic and septic shock. Therefore, the most likely explanation for persistent type B lactic acidosis in our patient appeared to be from DLBCL.

## Conclusions

Although elevated LA in ICU is common, our case emphasizes the fact that not all lactate production is due to tissue hypoxia or ischemia and that other sources should also be evaluated. Treatment of the underlying malignancy along with possible thiamine administration appears to be common management strategies. However, in the absence of well-defined treatment protocols, more research is necessary to understand and manage malignancy-related type B lactic acidosis.
